# A Spatial Analysis of Individual- and Neighborhood-Level Determinants of Malaria Incidence in Adults, Ontario, Canada

**DOI:** 10.3201/eid1805.110602

**Published:** 2012-05

**Authors:** Rose Eckhardt, Lea Berrang-Ford, Nancy A. Ross, Dylan R. Pillai, David L. Buckeridge

**Affiliations:** McGill University, Montreal, Quebec, Canada (R. Eckhardt, L. Berrang-Ford, N.A. Ross, D.L. Buckeridge);; University of Toronto, Toronto, Ontario, Canada (D.R. Pillai);; Ontario Agency for Health Protection and Promotion, Toronto, Ontario, Canada (D.R. Pillai);; Agence de la Santé et des Services Sociaux de Montréal, Montreal (D.L. Buckeridge)

**Keywords:** Malaria, health status disparities, immigration, Ontario, Canada, travel, spatial distribution, geography, medical, surveillance, Plasmodium vivax, Plasmodium falciparum, vector-borne infections, parasites

## Abstract

Malaria, once endemic in Canada, is now restricted to imported cases. Imported malaria in Canada has not been examined recently in the context of increased international mobility, which may influence incidence of imported and autochthonous cases. Surveillance of imported cases can highlight high-risk populations and help target prevention and control measures. To identify geographic and individual determinants of malaria incidence in Ontario, Canada, we conducted a descriptive spatial analysis. We then compared characteristics of case-patients and controls. Case-patients were significantly more likely to be male and live in low-income neighborhoods that had a higher proportion of residents who had emigrated from malaria-endemic regions. This method’s usefulness in clarifying the local patterns of imported malaria in Ontario shows its potential to help identify areas and populations at highest risk for imported and emerging infectious disease.

## Abstract 2

Imported malaria cases in adults are strongly patterned by neighborhood economic and immigration levels.

Malaria is a parasitic, vector-borne disease that causes ≈1 million deaths each year and substantial global public health costs (*1**,**2*). The disease was previously endemic in North America, with transmission in most of the United States and parts of southern Canada (*3*). The malaria parasite, *Plasmodium* spp., was introduced into North America during the 16th–17th centuries through the arrival of European colonists and African slaves (*3*). Malaria was eliminated in North America by the 1950s through several different interventions, including vector control by changes in vector habitat, introduction of new antimalarial medications (such as quinine) for improved treatment, and decreased contact between humans and mosquitoes, in part because of changes in housing conditions (*3*).

Cases of locally acquired, mosquito-transmitted (autochthonous) malaria still occur in the United States, particularly in the Northeast. Most recently, reported autochthonous cases in the United States have occurred in suburban or urban areas (*3*). Although no confirmed cases of autochthonous malaria have been recorded in Canada in recent years, ≈400 cases of imported malaria are identified in Canada each year, a significantly higher prevalence than in the United States (*4**,**5*). Increased international travel and immigration have the potential to change the probability of autochthonous transmission in Canada and the United States, where competent vectors and suitable regional climates already exist (*3**,**6*).

Malaria incidence is affected by socioeconomic inequality and human movement (*7*). Malaria transmission in Canada was associated historically with socioeconomic inequality and migration; in particular, malaria transmission surged among migrant workers on the Rideau Canal in Ontario during 1826–1832 (*8*). Regional and international travel and migration patterns have also been implicated in malaria incidence across the globe, including reports of so-called airport malaria, refugee outbreaks, and cases in migrant populations (*9**–**12*). Rates of transmission in highly traveled areas have been found to affect rates of imported malaria cases (*13*). Length and type of travel and travel behavior are associated with risk for malaria transmission (*14**–**16*). At particularly high risk of acquiring malaria and importing it to their country of residence are travelers who visit friends and relatives (VFRs) in countries where malaria is endemic (*16**–**21*). Research has recently shown that VFRs are also at risk for multidrug-resistant malaria because of the inappropriate use of antimalarial drugs available over the counter in malaria-endemic countries (*22*).

Emerging and reemerging disease risk interacts with socioeconomic vulnerability to determine the populations at highest risk for infection and those most likely to import pathogens into the country. In the context of changing patterns in international travel, immigration, and global disease spread, understanding the socioeconomic determinants of existing disease risks is prudent.

In this study, we first conducted a descriptive spatial analysis to identify the geographic and individual determinants of malaria incidence in Ontario, Canada. We then tested the hypothesis that malaria case-patients do not differ significantly from controls in terms of their individual and geographic characteristics.

## Methods

### Study Design

The study location was Ontario, Canada. Data on positive and negative malaria tests conducted in Ontario were used to assign persons to 1 of 2 groups: case-patients (persons who tested positive for *Plasmodium* spp. by standard microscopy using Giemsa stain) and controls (persons who tested negative for *Plasmodium* spp.). First, descriptive statistics, mapping, and space–time cluster analysis were used to examine the spatial patterns of malaria incidence in Ontario and associated individual and geographic risk factors. Second, logistic regression was used to test the hypothesis that malaria case-patients and controls did not differ significantly in their individual and geographic determinants.

### Location Context

Toronto, the largest city in the province and country, experiences a high volume of international travel, with 3.5 million passengers arriving at Toronto’s Pearson International Airport in 2007 (*23*). Toronto is a major immigration destination, globally and within Canada (*24*). One fifth of Canadians were born outside of Canada, and in the census metropolitan area of Toronto, the proportion is more than twice the national rate, with 45.7% of the population born outside Canada (*25*). Locations where immigrants choose to settle within the greater Toronto area (GTA) show the strongest growth of cities outside the city of Toronto (*25*).

### Data Sources

Data on malaria cases were obtained from the Malaria Reference Laboratory, Public Health Laboratories (PHL), Ontario Agency for Health Protection and Promotion (Toronto). These data included results of malaria tests conducted in the 12-month period from May 2008 through April 2009, and they identified patients as either positive (case-patients) or negative (controls) by reference thick and thin blood films. PHL receives blood specimens from other laboratories throughout the province and from hospitals and clinics. The data include the results of any test conducted at PHL for malaria diagnosis, confirmation, or malaria parasite speciation. PHL conducts ≈75% of the malaria testing for Ontario. An ethics certificate was obtained from the McGill University Research Ethics Board, and the de-identified data were stored in a confidential and secure manner.

The dataset from the Ontario Agency for Health Protection and Promotion contained 990 specimens. A specimen represented a blood sample that was tested for malaria. Specimens from persons for whom home postal code information was not available and those for whom home addresses were outside of Ontario were excluded, leaving 770 specimens. Data were converted from specimens to observations (persons) to avoid duplication in the dataset because some persons had results for multiple tests. Conversion from specimen to person was based on home postal code, birth date, and sex. This conversion resulted in 562 observations (persons). Malaria can be more severe in children, and symptoms in children are similar to those of other diseases (*26**,**27*); these facts may prompt a higher index of suspicion and different patterns of diagnostic testing for children and adults. The dataset included many negative test results for children, and combined with a low number of child case-patients, stratified analysis of malaria in children was not feasible; persons <18 years of age were thus excluded.

The final dataset included 354 observations of individual adults who tested positive or negative for *Plasmodium* spp. These persons were categorized as case-patients (n = 94) and controls (n = 260). A travel history was reported for 151 of these persons. Data on immigration status were not available. Such information would have been a valuable component of the analysis.

Census data describing population size, residents’ immigration status, and median household income were compiled for all Ontario census tracts. Census tracts were used as a proxy for neighborhoods (*28*), and population density was calculated for each area. Proportion of residents who are immigrants from malaria-endemic countries for each neighborhood was calculated by dividing the number of respondents reporting immigration from an area where malaria is endemic (Southeast Asia, South Asia, East Africa, Central Africa, West Africa) by the number of census respondents. Geographic boundaries of both census tracts (population size ≈2,000–8,000 persons) and dissemination areas (population size ≈400–700 persons) were used for mapping, area calculation, and spatial reference.

### Descriptive Analysis

All cartography was carried out by using ArcGIS version 9.3 software (ESRI, Redlands, CA, USA). Several key variables were used in the descriptive mapping of malaria case-patients: case-control status, parasite species, and age and sex of participant. For case–control data, observations were placed at the geographic center of the postal code recorded as the home address. Six-digit postal codes were used for all observations retained in the dataset, and those without appropriate postal codes were removed. Placing the observation at the geographic center of the postal code ensured accurate representation of location while maintaining participant privacy. Week codes (a numerical designation by the US Centers for Disease Control and Prevention for each week of the year, 1–52) were assigned to observations in the dataset to facilitate temporal graphing. Timeline graphs were created for evaluating the seasonal and temporal distribution of malaria observations. Incidents were stratified by parasite species.

Travel history was categorized by using the regional groupings provided in the 2006 Canadian census. Univariate tests and graphing were performed for the variables of travel, neighborhood immigration characteristics, case or control status, and parasite species. For travel and immigration analysis, observations that noted a parasite species other than *P. falciparum* and *P. vivax* (n = 9) were excluded.

### Cluster Analysis

Because of a high concentration of cases in the GTA, a subset of the malaria case dataset was created, with only observations in the GTA. For purposes of analysis, Canadian regional municipalities were used to delimit the dataset, and the GTA subset thus included Toronto, Halton, Peel, York, and Durham municipalities. Cluster analysis was performed on the GTA subset to assess the significance of any potential spatial clustering. A space–time scan statistic (*29*) was performed on case–control status and parasite species by using SaTScan version 8.1.1 software (M. Kulldorff, Boston, MA, USA). The scan statistic compares observed case counts within a range of alternate scan windows, in both space and time, to expected case counts derived from Monte Carlo random simulation. The scan window with the maximum likelihood ratio statistic is identified and compared with the null hypothesis of no significant clustering. Analyses assumed a binomial distribution in which the 2 possible outcomes were to be either a case-patient or a control. The maximum cluster size was 50%, so that no more than 50% of the observations could be classified as being part of the detected cluster.

### Logistic Regression

The key variables in the logistic regression model were population density, median household income, proportion of residents who are immigrants from malaria-endemic countries, and sex. Predictor variables were chosen on the basis of individual and ecologic risk factors for travel-related disease and infectious disease. The outcome variable was case–control status. All statistical analyses were assessed for significance at the 95% CI. Univariate tests were conducted to assess the relationship between the individual predictor variables and case–control status. One observation was removed because no census data were available for the postal code geographic center. All variables were checked for co-linearity and normality, and the model was checked for notable interaction, outliers, confounding, predictive ability and accuracy, leverage, and goodness-of-fit. The best-fit model was chosen on the basis of the inclusion of the key variables related to imported malaria cases, given data availability and the results of the tests for goodness-of-fit and other postestimation procedures. The best-fit model was interpreted by using quartile values of each of the predictor variables, comparing the first and fourth quartiles. All statistical analyses were performed by using Stata version 11 software (StataCorp, College Station, TX, USA).

## Results

**Figure 1:**
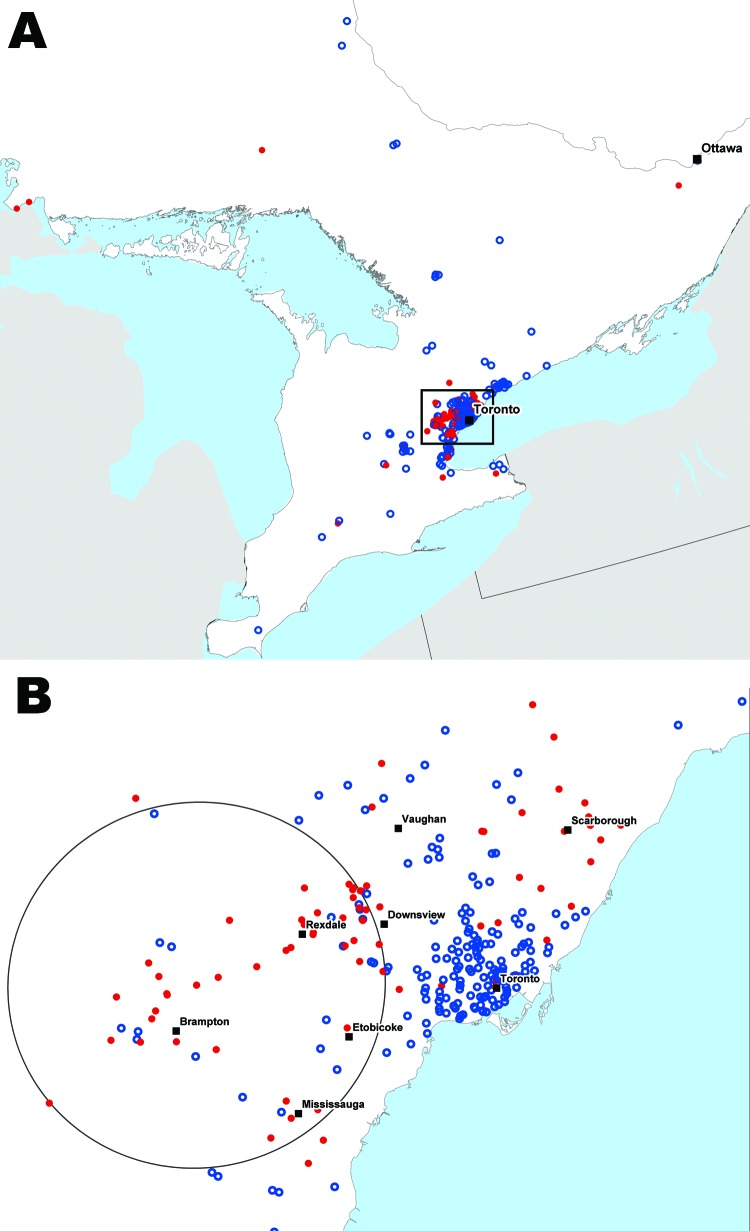
Locations of persons from whom 990 blood samples were taken and tested for malaria by the Ontario Agency for Health Protection and Promotion, Ontario, Canada, 2008–2009. Red dots, malaria case-patients (positive test results); blue circles, controls (negative test results). A) All observations; B) the most significant space–time cluster for malaria patients (circle), greater Toronto area, during May 15–November 6, 2008 (relative risk 3.54; p<0.01).

The general distributions of sex, age, and relative parasite proportions did not differ greatly between case-patients (n = 94) and controls (n = 260) for all of Ontario and the GTA subset (Table 1). The spatial distribution of the case-patients and controls was consistent with Ontario’s settlement patterns, with most of the observations located in southern Ontario and the GTA (Figure 1). The downtown core of Toronto had a large concentration of controls, whereas more case-patients lived in suburban areas.

**Table 1:** Summary characteristics of imported malaria case-patients and controls, Ontario, Canada, 2008–2009

Characteristic	Case-patients	Controls	Total
Ontario	94 (27)	260 (73)	354 (100)
Sex, no. (%)			
M	65 (33)	131 (67)	196 (100)
F	23 (15)	129 (85)	152 (100)
Not available	6 (100)	0	6 (100)
Mean age, y	42.5	41.3	41.7
Species, no. (%)			
* Plasmodium falciparum*	54		
* P. vivax*	31		
* P. ovale*	4		
*Plasmodium* spp. (unidentified )	3		
*Plasmodium* spp. (mixed)	1		
*Babesia* spp.	1		
Greater Toronto area subset	84 (29)	209 (71)	293 (100)
Sex, no. (%)			
M	58 (37)	98 (63)	156 (100)
F	20 (15)	111 (85)	131 (100)
Not available	6 (100)	0	6 (100)
Mean age, y	42.5	40.6	41.2
Species, no. (%)			
* P. falciparum*	48		
* P. vivax*	30		
* P. ovale*	3		
*Plasmodium* spp. (unidentified)	2		
*Plasmodium* spp. (mixed)	1		
*Babesia* spp.	0		

Significant clustering of case-patients occurred in suburban Toronto during the summer months (Figure 1). A significant space–time cluster was identified during May 15, 2008–November 6, 2008, in the region northwest of Toronto near Brampton (p<0.01; relative risk 3.54). This area is near the main international airport in the GTA. In this area during the summer months, tested persons were 3.5× more likely to receive a diagnosis of malaria than were persons outside of this area and period. No significant clustering of individual parasite species (*P. falciparum* or *P. vivax*) was identified.

**Figure 2:**
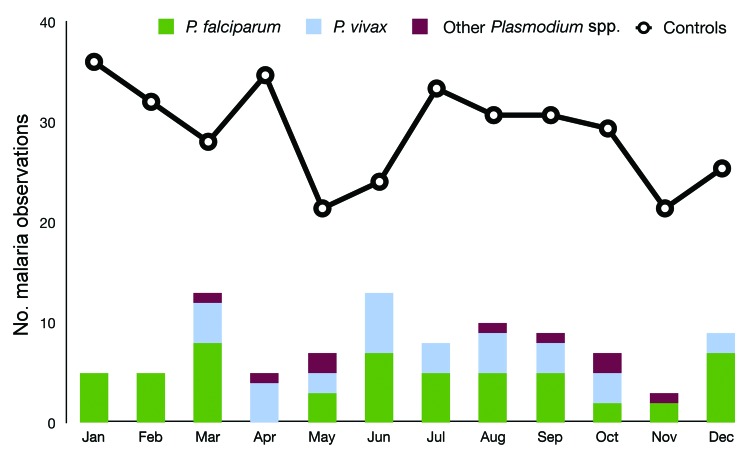
Case-patients, by *Plasmodium* species with which infected, and controls who tested negative for *Plasmodium* spp., by month, Ontario, Canada, 2008–2009.

Cases were not significantly more likely to occur in the summer months (June–September) than in the nonsummer months for the GTA and for all of Ontario (χ^2^; p = 0.14 and p = 0.11, respectively). This result is consistent with that of the space–time cluster analysis, which found that the significant space–time cluster extended through summer into autumn. Visual observation of monthly incidence, however, did not indicate any strong or clear trends, possibly because of the influence of low observation numbers, and incidence and seasonality in malaria-endemic countries from which the disease is imported (Figure 2).

**Figure 3:**
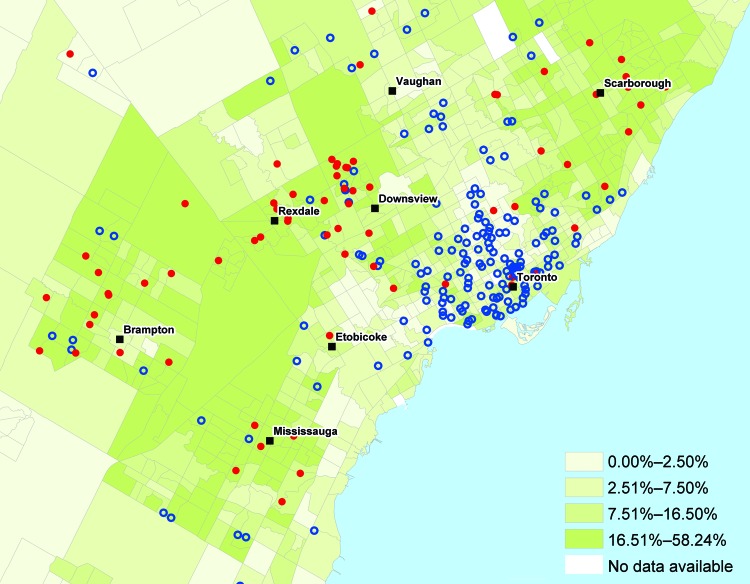
Percentage of residents in a neighborhood reporting immigration from malaria-endemic areas, greater Toronto area, Ontario, Canada, 2008–2009. Red dots, malaria case-patients (positive test results); blue circles, controls (negative test results).

**Figure 4:**
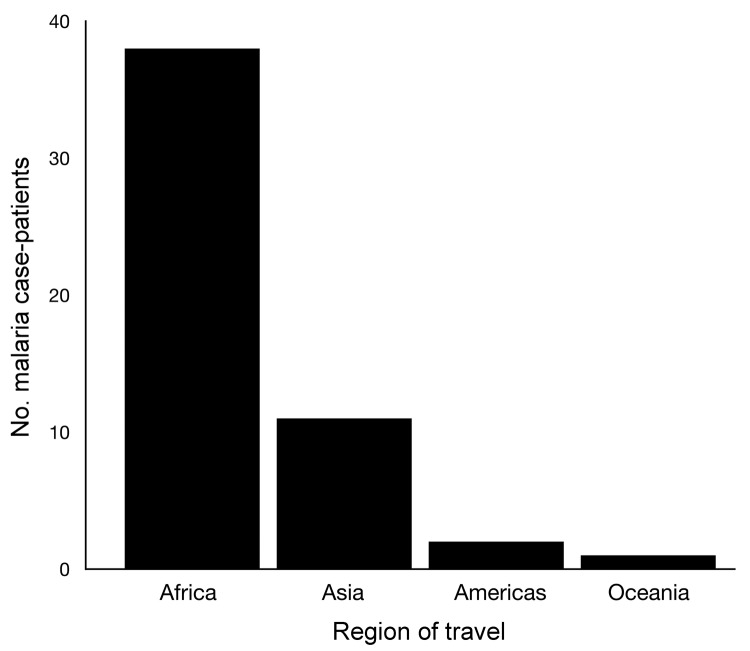
Number of malaria case-patients by region of travel, Ontario, Canada, 2008–2009.

Population density was highly variable in the study area, with more densely populated areas located near the downtown core of the GTA. Median household income, in contrast, exhibited negligible clustering except for low-income suburbs east and west of the downtown core. The proportion of residents who were immigrants from malaria-endemic regions was highest in the suburban areas to the east and west of Toronto (Figure 3). More than 94% of case-patients for whom travel history was recorded indicated recent travel to Africa or Asia (Figure 4), with travel to Africa reported most frequently. These results are consistent with known spatial ranges of malaria-endemic areas (*30*).

Case-patients were significantly more likely than controls to be male and live in low-income neighborhoods with a higher proportion of residents who immigrated from malaria-endemic regions (Table 2). The proportion of residents who were immigrants from malaria-endemic countries was, for example, >2× as high in neighborhoods with case-patients than in neighborhoods where controls were identified. The average income of case-patient neighborhoods was ≈$28,000, compared with ≈$31,000 in control neighborhoods (Student *t* test, p = 0.04). Case-patients with a travel history (n = 47) were more likely to report having traveled to Africa and to be living in a neighborhood with a higher proportion of residents who are immigrants from malaria-endemic countries than controls with a travel history (Table 3).

**Table 2:** Univariate analysis of malaria case-patients and controls, Ontario, Canada, 2008–2009

Variable	Case-patients	Controls	p value
Total, no. (%)	94 (100)	259 (100)	
Individual level			
Sex, no. (%)			
M	65 (69)	130 (50)	<0.01*
F	23 (25)	129 (50)	
Not reported	6 (6)	0	
Age, mean (95% CI)	42.5 (39.4–45.7)	41.4 (39.3–43.5)	0.57†
Neighborhood-level, mean (95% CI)			
Population density, persons/km	4,633 (3,926–5,340)	6,498 (5,634–7,363)	0.12‡
Median income (Canadian dollars)	28,140 (26,232–30,047)	30,802 (29,416–32,187)	0.04†
Residents who are immigrants from malaria-endemic areas, %	18.1 (15.5–20.7)	8.8 (7.6–10.0)	<0.01‡

*χ^2^ test. †Student *t* test. ‡Mann-Whitney U test.

**Table 3:** Univariate analysis of travel and immigration and case–control and parasite species variables, Ontario, Canada, 2008–2009

Variable	Patients				Parasite species		
	Case-patients	Controls	p value		*Plasmodium falciparum*	*P. vivax*	p value
Neighborhood-level, mean %							
Residents who are immigrants from malaria-endemic Africa	3.1	1.5	<0.01*		3.8	2.0	0.33*
Residents who are immigrants from malaria-endemic Asia	15.5	7.2	<0.01*		12.4	20.8	<0.01*
Individual level, no.							
Travel to malaria-endemic Africa	36	27	<0.01†		35	1	<0.01‡
Travel to malaria-endemic Asia	11	25	0.61†		0	11	<0.01‡

*Mann-Whitney test. †χ^2^ test. ‡Fisher exact test.

The regression results are consistent with univariate analyses: case-patients were more likely to be male (odds ratio [OR] 2.24, 95% CI 1.24–4.05) and live in neighborhoods with high levels of immigration from malaria-endemic countries (OR 1.09, 95% CI 1.06–1.12) (Table 4). Population density was a weak predictor, but was retained in the model to account for potential confounding with proportion of residents who are immigrants from malaria-endemic regions of Africa. This variable had a wide range (0%–58%) and showed the strongest effect on case status: for every 1% increase in the proportion of residents who are immigrants from malaria-endemic regions, the odds of being a case-patient increased by 7%. The odds of being a case-patient were >17× higher in neighborhoods in the top quartile of proportion of residents who are immigrants from malaria-endemic areas than in the neighborhoods in the lowest quartile. There were slight negative effects from median household income and population density. For every 1 unit increase in either, the odds of being a case-patient decreased by 1%.

**Table 4:** Results of regression analyses of individual- and neighborhood-level variables of malaria incidence, Ontario, Canada, 2008–2009*

Variable	Odds ratio (95% CI)		
	Null model	Fully adjusted	1st vs. 4th quartile
Residents who are immigrants from malaria-endemic areas, %	1.07 (1.05–1.09)	1.09 (1.06–1.12)	17.66 (7.17–43.48)
Median income, CAD$	0.99 (0.99–0.99)	0.99 (0.99–0.99)	3.29 (1.34–8.09)†
Population density	0.99 (0.99–0.99)	0.99 (0.99–0.99)	6.39 (2.48–16.49)†
Male sex	2.80 (1.64–4.78)	2.24 (1.24–4.05)	NA

*Dependent variable: case-patients (positive malaria test); goodness-of-fit, Hosmer-Lemeshow test, p = 0.76. CAD$, Canadian dollars; NA, not applicable. †Reverse coded to show 4th vs. 1st quartile.

The pseudo R^2^ of the model was 0.19, with a predictive accuracy of 70% correctly classified by using a cutoff of 0.25 (67% sensitivity, 71% specificity). The model fit the data well (Hosmer-Lemeshow test, p = 0.76), and leverage values did not indicate any outliers that required removal. Several high positive residuals were found, because of the low sensitivity of the model that used a default cutoff (0.5). This low sensitivity reflects the limited number of variables available for analysis. The model was developed, however, for explanatory rather than predictive purposes; low sensitivity reflects the small number of covariates and use of neighborhood-level variables.

The distribution of parasite species infection in case-patients reflects the patterning of global malaria; *P. vivax* cases are associated with travel to or immigration from Asia, and *P. falciparum* cases are associated with travel to Africa (Table 3). Globally, a significant spatial patterning of parasite species exists, with *P. falciparum* found predominantly in Africa and *P. vivax* found predominantly in Asia (*30*). Case-patients infected with *P. falciparum*, for example, were significantly more likely to report recent travel to Africa than case-patients infected with *P. vivax* (Fisher exact test, p<0.01). Conversely, case-patients infected with *P. vivax* were more likely to report travel to malaria-endemic regions of Asia (Fisher exact test, p<0.01). Case-patients with *P. vivax* lived in neighborhoods with a significantly higher proportion of residents who are immigrants from malaria-endemic regions (Mann-Whitney, p<0.01). In contrast, however, *P. falciparum* case-patients did not necessarily live in neighborhoods with a high proportion of residents who are immigrants from malaria-endemic regions of Africa (Mann-Whitney, p = 0.33). This finding may be due to the much lower overall level of immigration from Africa.

## Discussion

Malaria case-patients in Ontario were found predominantly in the GTA, with more case-patients in suburban areas outside the city center. Malaria case-patients were more likely to live in neighborhoods with a high proportion of residents who emigrated from malaria-endemic areas. Case-patients were more likely than controls to report travel to areas with endemic malaria, and there was concordance between the parasite species and the region of travel. The association between parasite species and geography held when neighborhood immigration was examined as well—cases of *P. vivax* malaria corresponded with immigration from malaria-endemic areas of Asia, and cases of *P. falciparum* malaria were found in areas with immigration from malaria-endemic areas of Africa.

Case-patients were more likely to be male than female. This finding is consistent with the literature on sex and travel-associated diseases, which finds that men are more likely than women to have vector-borne diseases, including malaria (*31*). Whether this finding is due to a biologic predisposition or to behavioral differences associated with increased transmission risk is not known (*31*). Whether potential differences in patterns of health care use between men and women could have affected the results in this study also is not known. Further research into how men and women seek pretravel and posttravel medical care in Ontario would improve the understanding of this result.

When assessing imported malaria cases, underreporting is a substantial issue. In Ontario, underreporting of malaria is likely to range from 10% to 40% (*5**,**32*). In Ontario, 226 malaria cases were documented by laboratories in 1998 (*32*). However, in that same year, only 160 cases in Ontario were reported to Health Canada (*32*). No recent published or quantified estimates of actual incidence are available, and the true level of current underreporting of malaria in Ontario and Canada more generally remains unclear. The data used in the study are strengthened by the inclusion of negative and positive malaria test results. The use of controls enables the factors that correlate with malaria cases to be better understood, unbiased by underlying patterns of malaria testing.

The odds of becoming infected and importing malaria to Canada were >17× higher for residents of neighborhoods with high immigration from malaria-endemic areas than those with low immigration from malaria-endemic countries. These results, combined with the geographic findings of the cluster analysis, are supported by census data on immigration. Brampton (the location of the significant space–time cluster of malaria cases), Markham, and Ajax all showed a significant increase in the proportion of residents born outside Canada in the 2006 census (*25*). As immigration to the neighborhoods in the suburbs of the GTA grows, more imported malaria cases could result. These results have implications for potential preventative measures that could be taken before persons travel abroad. The use of cluster detection methods has been proposed in other areas to target prevention methods (*33**,**34*). Targeted prevention could focus on hospitals and clinics found in the cluster area and in regions with a high proportion of residents who are immigrants from malaria-endemic areas, with the goal of implementing targeted travel medicine screenings and education regarding malaria prophylaxis.

Predeparture travel medicine services are not covered under Ontario’s provincial insurance plan (*35*). However, the use of travel clinics is not associated with income in Canada (*36*). As a high-risk group, VFRs are characterized by differing preventive care choices and lower use of pretravel medical services (*37*). In Canada, 1 study found that VFRs consulted family practitioners more often than travel clinics and that the family practitioners were more likely to prescribe inappropriate chemoprophylaxis (*38*). Another reason for the increased risk for imported malaria among VFRs could be differing perceptions of the risk for malaria, leading to different behavior in malaria-endemic areas (*16**,**21**,**39*). The recent growth of immigrants in Brampton and related Toronto suburbs may merit targeted programming by physicians and public health agencies, including translation of public health prevention materials (*25*).

Imported malaria in Canada is of public health significance given current, and likely substantially underestimated, cases of imported disease in Canada. Our characterization of imported malaria cases in Ontario highlights increased risk for infection in immigrant and low-income neighborhoods, with associations between immigration from countries where malaria transmission and particular pathogen types are endemic. These results suggest a pattern of inequality in tropical health outcomes in Ontario with implications for prevention and control of current incidence.

Imported malaria cases represent the potential for geographic emergence of a range of travel-associated diseases. The current landscape of emerging infectious diseases demands that we improve our understanding of the mechanisms by which diseases are imported and the characteristics of neighborhoods and populations at highest risk. The results of this study point to potential individual- and neighborhood-level risk factors that might be relevant to emerging infectious diseases more generally. As new infectious diseases emerge, identifying the processes of emergence and the areas and persons at greatest risk is critical. An analysis of the local patterns of imported malaria in Ontario can help the public health community better understand the ways in which global travel and immigration can affect the spatial distribution of other travel-related diseases.
